# Enhanced Nitrogen Supply Sustains Post-Silking Canopy Function and Grain Yield of Drip-Irrigated Spring Maize Under Increased Planting Density in Northern Xinjiang

**DOI:** 10.3390/plants15213467

**Published:** 2026-11-06

**Authors:** Qi Xu, Changkun Ma, Quanjiu Wang, Qiuping Fu

**Affiliations:** 1 State Key Laboratory of Water Engineering Ecology and Environment in Arid Area, Xi’an University of Technology, Xi’an 710048, China; 2 Institute of Arid Region Ecological Water Conservancy, Xi’an University of Technology, Xi’an 710048, China; 3 College of Hydraulic and Civil Engineering, Xinjiang Agricultural University, Urumqi 830052, China

**Keywords:** spring maize, planting density, nitrogen rate, photosynthesis, dry matter translocation

## Abstract

How nitrogen (N) supply should be matched to increased planting density in drip-fertigated maize in arid regions remains unclear. A two-year split-plot field experiment (2024–2025) at Shihezi, northern Xinjiang, China, tested two planting densities (D1, 60,000; D2, 90,000 plants ha^−1^) and two N rates (N1, 180; N2, 240 kg N ha^−1^) in film-mulched, drip-irrigated spring maize. D2 increased grain yield by 13.9% over D1, because a 46.8% increase in ear number outweighed decreases in kernels per ear and kernel weight. The yield response to N depended on density (D × N, *p* < 0.01): N2 increased yield by 13.8% under D2 but by only 3.6% (not significant) under D1. N2 increased N accumulation at silking similarly at both densities, whereas its post-silking effects were much larger under D2, where it reduced the post-silking decline in leaf area index from 67.3% to 51.2% and increased post-silking dry matter accumulation and apparent N uptake by 22.0% and 44.3%. D2N2 produced the highest yield (11.51 t ha^−1^), whereas D2N1 achieved the highest partial factor productivity of N (56.2 kg kg^−1^). At this single site, dense planting benefited more from the higher N rate, most evidently after silking; validation across sites, hybrids, and N rates is needed.

## 1. Introduction

Maize (*Zea mays* L.) is the cereal with the largest global production and a major source of food, feed, and industrial raw materials [1,2]. In China, arable land can hardly be expanded, so further gains in maize output must come from higher yield per unit area, and considerable yield gaps still exist between farmers’ fields and attainable yields [3,4]. The irrigated oasis agriculture of Xinjiang, northwest China, has a particularly high yield potential owing to abundant solar radiation, a large diurnal temperature range, and irrigation; the highest maize yields recorded in China, exceeding 21 t ha^−1^, have been obtained in this region under dense planting [5,6]. Because annual precipitation in northern Xinjiang is only about 200 mm, maize there is widely grown with drip irrigation under plastic film mulch, a system that allows water and fertilizer to be supplied together through fertigation [7,8].

Increasing planting density has been a major driver of historical maize yield gains, because modern hybrids tolerate crowding better than older ones and produce more ears per unit area [9,10,11,12]. A recent global meta-analysis showed that higher density increases population grain yield mainly through greater pre-silking dry matter (DM) production and its remobilization, whereas per-plant DM production declines more strongly after silking than before [13]. As density increases, competition among plants for light, water, and nutrients reduces per-plant leaf area and growth rate around silking, increases plant-to-plant variability, and decreases kernel number and kernel weight [14,15,16,17]. Dense canopies are also more prone to lodging [18]. Because most grain DM in modern hybrids is produced after silking [19,20], the benefit of dense planting largely depends on whether the canopy can maintain its photosynthetic capacity during grain filling. Consistent with this, record-yielding maize in Xinjiang grown at 120,000–135,000 plants ha^−1^ combined a leaf area index of about 7.5 at silking with post-silking DM accumulation of about 20 t ha^−1^ [5].

Nitrogen (N) is the nutrient that most strongly limits maize productivity; it regulates leaf area expansion, radiation use, and kernel set [21,22]. Higher densities increase population N demand, and plant N uptake per plant declines under crowding [23]. Field evidence from intensive maize systems in China indicates that high densities with crowding stress reduce the ability of maize to use soil N after silking, so that a higher N rate is needed to realize the yield potential of dense stands [24]. Conversely, N fertilizer is commonly overused in intensive Chinese cropping systems, which lowers N use efficiency (NUE) and increases reactive N losses to the environment [25,26,27]. Matching N supply to population demand is therefore essential to achieve high yield and high NUE simultaneously [28]. Drip fertigation offers a practical means of doing so, because N can be applied in several split doses with irrigation water and timed to crop demand, including during grain filling [29,30].

Several studies have combined increased density with reduced or optimized N rates and reported higher yield and NUE than conventional practice [31,32]. Under drip fertigation in northwest China, the yield response to N rate differed between densities, and the N rate required for near-maximum yield changed with density [30]; in high-yield fields in Xinjiang, the match between the vertical distributions of canopy N and light was closely related to yield and radiation use efficiency [33]. Higher N supply has also been shown to partly offset the decrease in kernel weight under high density [34]. However, most of these studies focused on yield, water productivity, and NUE, and the physiological processes through which N supply modifies the response to density remain insufficiently understood. In particular, it is unclear whether the larger N response of dense stands arises before silking, through a larger canopy and more stored N, or after silking, through delayed leaf senescence and sustained post-silking photosynthesis, DM accumulation, and N uptake [35,36,37]. This distinction is important for drip-irrigated maize in arid oases, where strong radiation and high evaporative demand during grain filling [7,38] may accelerate leaf senescence in crowded canopies.

The present study was designed to address this question. Rather than comparing only grain yield and NUE among density–N combinations, we quantified, in the same plots, the additional N uptake and DM accumulation obtained with the higher N rate before and after silking, so that the N response at each density could be separated into pre-silking and post-silking components. These population-level DM and N dynamics were measured together with leaf area dynamics and with ear-leaf gas exchange and SPAD value at silking and during grain filling, which allowed us to identify the growth stage at which the density-dependent N response became evident. The experiment was conducted under film-mulched drip fertigation in an arid oasis, where 80% of the N was split among six fertigation events from the sixth-leaf stage to the milk stage, so that N could still be supplied during grain filling, the period in which the per-plant DM production of dense stands is most strongly reduced [13].

We hypothesized that (i) increasing planting density raises grain yield mainly by increasing ear number, and (ii) the greater yield response to N under high density is associated mainly with post-silking processes, namely the maintenance of leaf area index (LAI), leaf photosynthesis, and N uptake, rather than with a larger pre-silking response to N. To test these hypotheses, a two-year field experiment with two planting densities and two N rates was conducted with drip-fertigated spring maize at Shihezi, Xinjiang. The objectives were to (1) quantify the effects of density, N rate, and their interaction on grain yield and its components; (2) characterize the responses of LAI, leaf photosynthesis, and DM and N accumulation and translocation; and (3) compare grain yield and partial factor productivity of N among the four density–N combinations to identify options for different production objectives at this site.

## 2. Results

### 2.1. Analysis of Variance

Planting density (D) significantly affected all yield-related and physiological traits listed in Table 1a, and N rate (N) affected all traits except ear number and harvest index (HI). The D × N interaction was significant (*p* < 0.01) for grain yield, kernels per ear, post-silking DM accumulation, total N accumulation at maturity, and partial factor productivity of N (PFPN). For the canopy and ear-leaf traits (Table 1b), D significantly affected LAI and the net photosynthetic rate (*P*_n_), stomatal conductance (*g*_s_), and SPAD value of the ear leaf at all stages shown, and N significantly affected all of these traits except LAI at silking (R1). The D × N interaction was not significant for LAI, *P*_n_, or *g*_s_ at R1, but it was significant for SPAD value at R1, LAI at physiological maturity (R6), and *P*_n_, *g*_s_, and SPAD value at the milk stage (R3), indicating that the density dependence of the N effects was limited at silking (significant only for SPAD value) and became more pronounced during grain filling. Results for LAI at the sixth-leaf (V6), twelfth-leaf (V12), and R3 stages and for the intercellular CO_2_ concentration (*C*_i_) are given in Table S2. Year (Y) had no significant effect on these traits, and none of the interactions involving year was significant. Therefore, treatment effects are described below using two-year means, while data for each year are shown in the tables and figures.

### 2.2. Grain Yield and Yield Components

Grain yield ranged from 9.20 t ha^−1^ (D1N1) to 11.41 t ha^−1^ (D2N2) in 2024 and from 9.44 t ha^−1^ (D1N1) to 11.61 t ha^−1^ (D2N2) in 2025 (Table 2). Compared with D1 (60,000 plants ha^−1^), D2 (90,000 plants ha^−1^) increased grain yield by 13.9% on average. This gain resulted from a 46.8% increase in ear number, which outweighed decreases of 17.1% in kernels per ear and 6.7% in 1000-kernel weight. HI was slightly lower under D2 (0.486) than under D1 (0.496).

The effect of N rate on yield depended on density. Under D1, N2 (240 kg N ha^−1^) increased yield by only 3.6% compared with N1 (180 kg N ha^−1^), and the difference was not significant in either year. Under D2, N2 increased yield by 13.8% (from 10.12 to 11.51 t ha^−1^), and the difference was significant in both years. This increase was mainly attributed to more kernels per ear (+9.1%) and a higher 1000-kernel weight (+4.6%), whereas ear number was not affected by N rate. As a result, D2N2 yielded 19.2% more than D1N2 and 23.5% more than D1N1.

### 2.3. Leaf Area Index and Photosynthetic Characteristics

**Figure 1:**
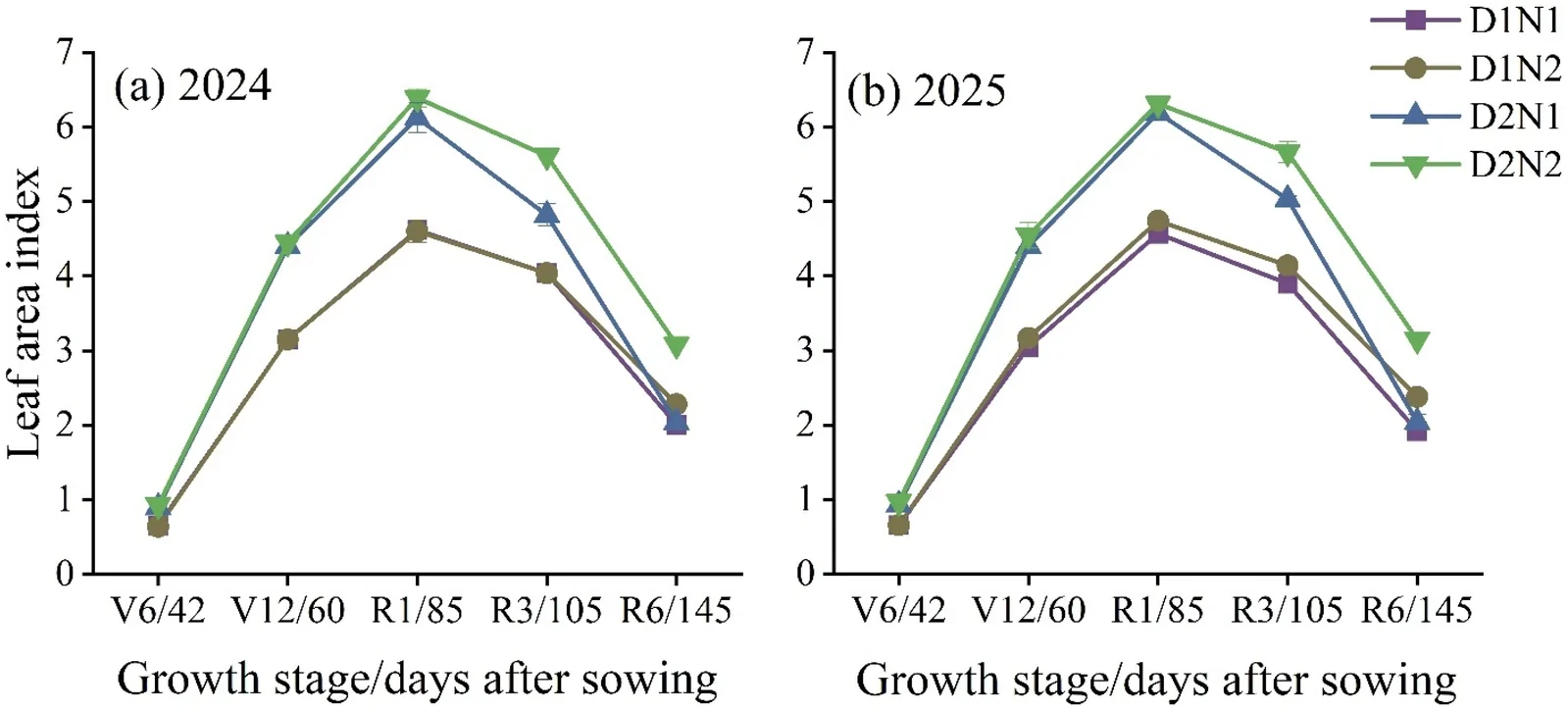
Dynamics of leaf area index (LAI) of spring maize under two planting densities and two nitrogen (N) rates in (**a**) 2024 and (**b**) 2025. V6, sixth-leaf stage; V12, twelfth-leaf stage; R1, silking; R3, milk stage; R6, physiological maturity. Numbers below the stage codes are days after sowing. D1 and D2, 60,000 and 90,000 plants ha^−1^; N1 and N2, 180 and 240 kg N ha^−1^. Vertical bars indicate standard errors (*n* = 3).

LAI increased until silking (R1) and declined thereafter in all treatments (Figure 1). Maximum LAI at R1 was 35.1% higher under D2 (6.25) than under D1 (4.63). N rate had little effect on LAI before silking; at V12, for example, LAI was 4.41 in D2N1 and 4.50 in D2N2. After silking, however, LAI declined faster in D2N1 than in the other treatments. From R1 to physiological maturity (R6), LAI decreased by 67.3% in D2N1 but only by 51.2% in D2N2, and by 57.2% and 50.4% in D1N1 and D1N2, respectively. At R6, LAI of D2N2 (3.10) was 54.2% higher than that of D2N1 (2.01).

**Figure 2:**
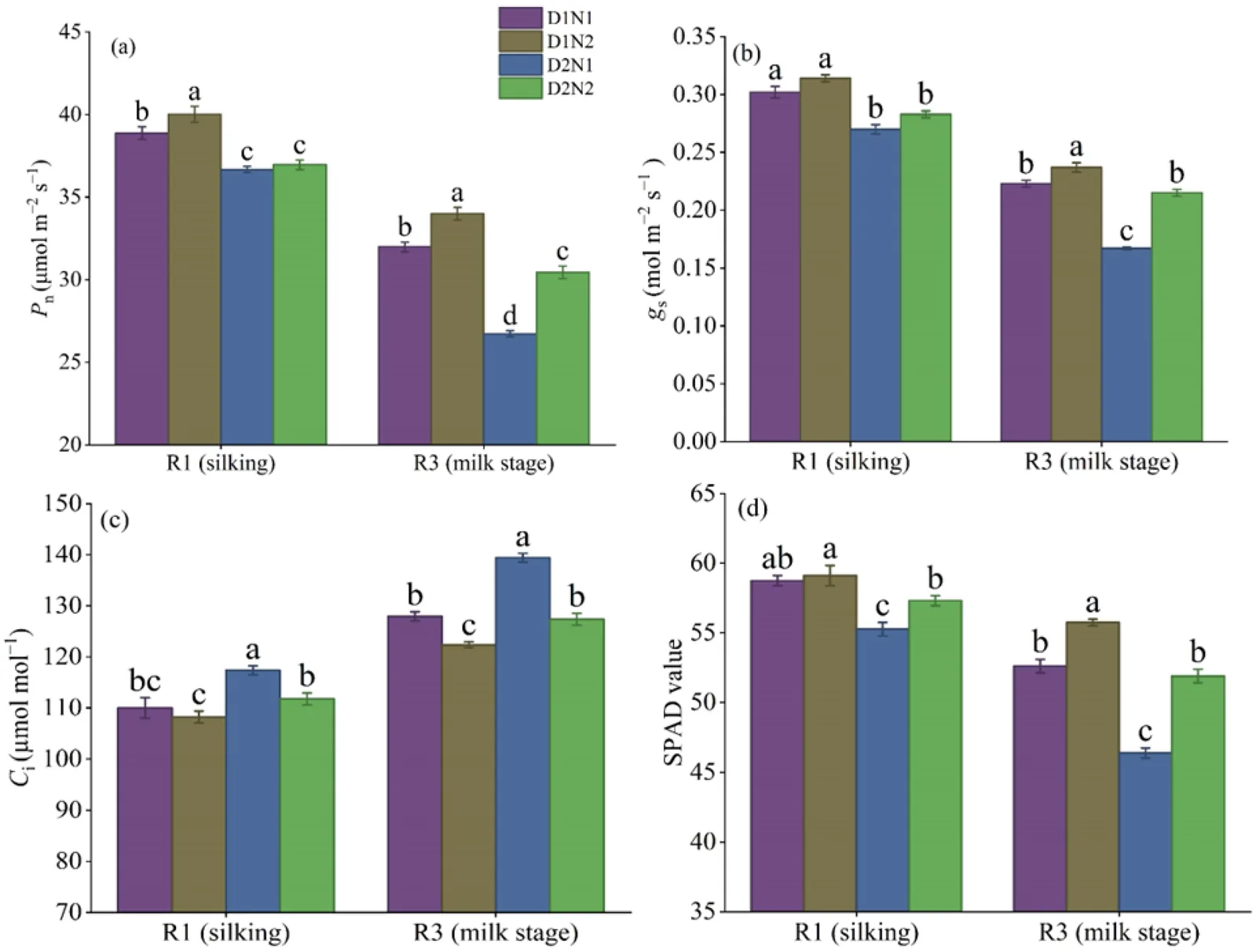
Net photosynthetic rate (*P*_n_) (**a**), stomatal conductance (*g*_s_) (**b**), intercellular CO_2_ concentration (*C*_i_) (**c**), and SPAD value (**d**) of the ear leaf at silking (R1) and the milk stage (R3) of spring maize under two planting densities (D1, 60,000 plants ha^−1^; D2, 90,000 plants ha^−1^) and two N rates (N1, 180 kg N ha^−1^; N2, 240 kg N ha^−1^). Values are means of two years ± standard errors (*n* = 6). Different lowercase letters above bars indicate significant differences among treatments at the same stage (LSD, *p* < 0.05).

At R1, the net photosynthetic rate (*P*_n_), stomatal conductance (*g*_s_), and SPAD value of the ear leaf were lower under D2 than under D1, and N rate had no significant effect on *P*_n_ or *g*_s_ under D2, although N2 already increased the SPAD value under D2 from 55.3 to 57.3 (Figure 2). Under D1, by contrast, N2 slightly but significantly increased *P*_n_ at R1 (Figure 2a). At R3, the differences among treatments became larger. *P*_n_ of D2N1 (26.7 µmol m^−2^ s^−1^) was 16.6% lower than that of D1N1 (32.0 µmol m^−2^ s^−1^), whereas N2 increased *P*_n_ by 14.1% under D2 and by 6.2% under D1. Similarly, N2 increased *g*_s_ and SPAD value at R3 under D2 by 28.0% and 12.0%, respectively. The intercellular CO_2_ concentration (*C*_i_) at R3 was highest in D2N1 (139.3 µmol mol^−1^), whereas it ranged from 122.5 to 128.0 µmol mol^−1^ in the other treatments.

### 2.4. Dry Matter Accumulation and Translocation

**Figure 3:**
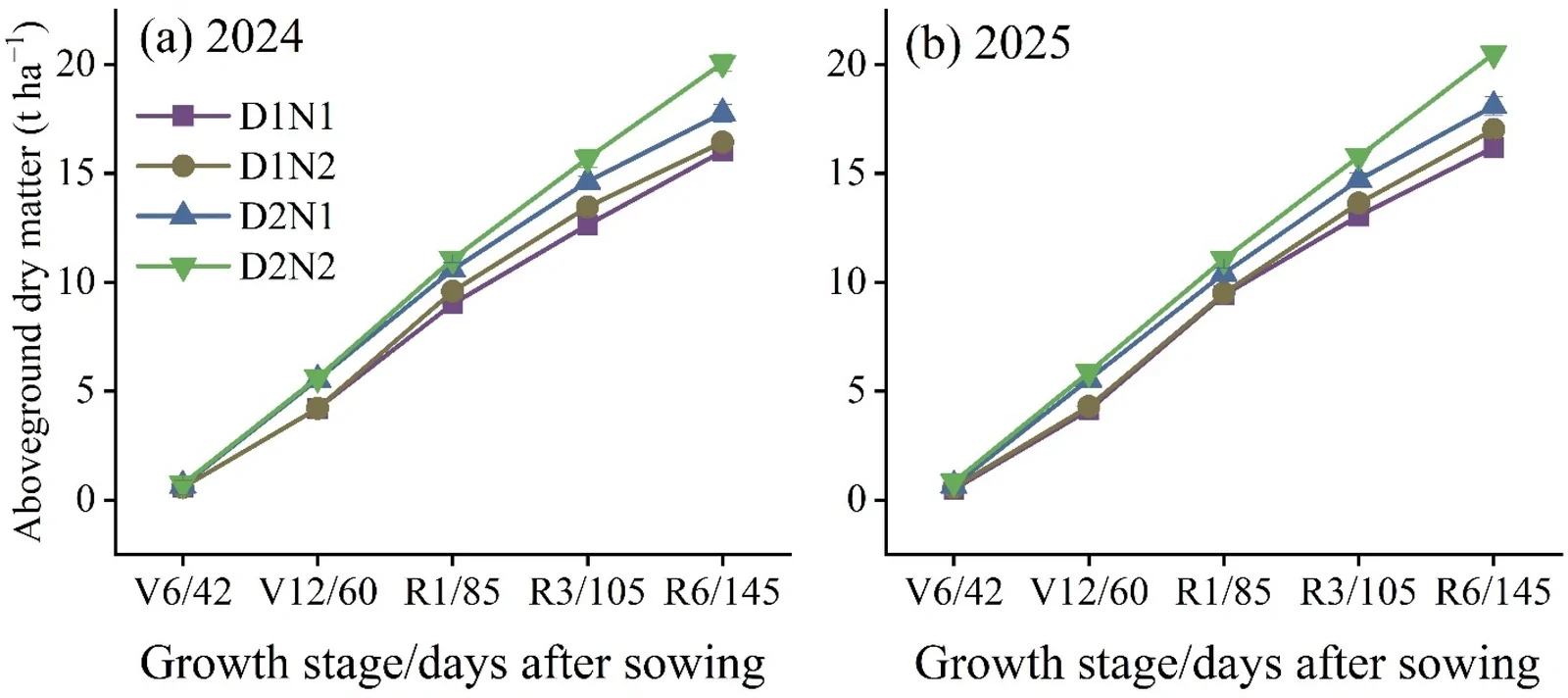
Aboveground dry matter accumulation of spring maize under two planting densities and two N rates in (**a**) 2024 and (**b**) 2025. Treatment and stage abbreviations are as in Figure 1. Vertical bars indicate standard errors (*n* = 3).

Aboveground DM increased throughout the growing season, and D2 accumulated more DM than D1 from V12 onwards (Figure 3). At R1, DM under D2 (10.82 t ha^−1^) was 15.7% higher than under D1 (9.35 t ha^−1^). Under D2, DM of D2N2 was 5.6% higher than that of D2N1 at R1 (11.11 vs. 10.53 t ha^−1^; significant only in 2025), and the difference increased to 12.4% at R6 (20.26 vs. 18.02 t ha^−1^).

Post-silking DM accumulation (PSDM) was highest in D2N2 in both years (Table 3). On average, PSDM in D2N2 (9.15 t ha^−1^) was 22.0% higher than in D2N1 (7.50 t ha^−1^), whereas N2 increased PSDM by only 3.1% under D1. Pre-silking DM translocation (DMT) ranged from 1.83 to 2.31 t ha^−1^ and did not differ significantly among treatments in either year. Post-silking assimilates therefore contributed most of the grain DM (CPSA, 71.8–80.3%). CPSA was numerically highest, and CDMT lowest, in D2N2 in both years, but the differences were significant only in 2025, when CPSA in D2N2 (80.3%) exceeded that in D1N1 (73.1%). These results suggest that the additional grain DM of D2N2 came mainly from current assimilation during grain filling rather than from greater remobilization of stored assimilates.

### 2.5. Nitrogen Accumulation, Translocation, and Use Efficiency

N accumulation at R1 and at R6 increased with both density and N rate (Table 4). N2 increased N accumulation at R1 by a similar amount under both densities: by 16.6 kg ha^−1^ (14.0%) under D1 (from 118.4 to 135.0 kg ha^−1^) and by 17.9 kg ha^−1^ (12.6%) under D2 (from 142.6 to 160.5 kg ha^−1^). Consequently, about 79% of the additional N uptake with N2 under D1 (about 21 kg ha^−1^) occurred before silking, whereas about 55% of that under D2 (about 40 kg ha^−1^) occurred after silking. N2 increased total N accumulation at R6 (Nt) by 12.1% under D1 (from 173.4 to 194.3 kg ha^−1^) and by 20.8% under D2 (from 192.9 to 233.0 kg ha^−1^). The larger response under D2 was mainly due to post-silking N uptake (PSNU), which was 44.3% higher in D2N2 (72.6 kg ha^−1^) than in D2N1 (50.3 kg ha^−1^), compared with an 8.0% increase under D1. Pre-silking N translocation (NT) tended to be higher under D2 than under D1 (60.5 vs. 52.2 kg ha^−1^, two-year means) but was similar between N rates under D2 (60.8 vs. 60.3 kg ha^−1^). The contribution of NT to grain N (CNT) was numerically highest in D2N1 in both years (53.1% and 56.0%), but differences among treatments were not significant. The N harvest index (NHI) ranged from 56.4% to 60.2% and was little affected by the treatments. Whereas N accumulation at R1 and R6 was measured directly, NT and PSNU are apparent values derived from the differences in N accumulation between these two stages (Section 4.4.3).

N2 decreased the N utilization efficiency (NUtE) by 7.4% under D1 and by 5.7% under D2. PFPN was highest in D2N1 (56.2 kg kg^−1^), followed by D1N1 (51.8 kg kg^−1^), D2N2 (48.0 kg kg^−1^), and D1N2 (40.2 kg kg^−1^), and the ranking was consistent across years. At the same N rate, D2 increased PFPN by 8.5% at N1 and by 19.2% at N2. Increasing the N rate decreased PFPN by 22.3% under D1 but by only 14.7% under D2.

### 2.6. Relationships Between Grain Yield and Physiological Traits

**Figure 4:**
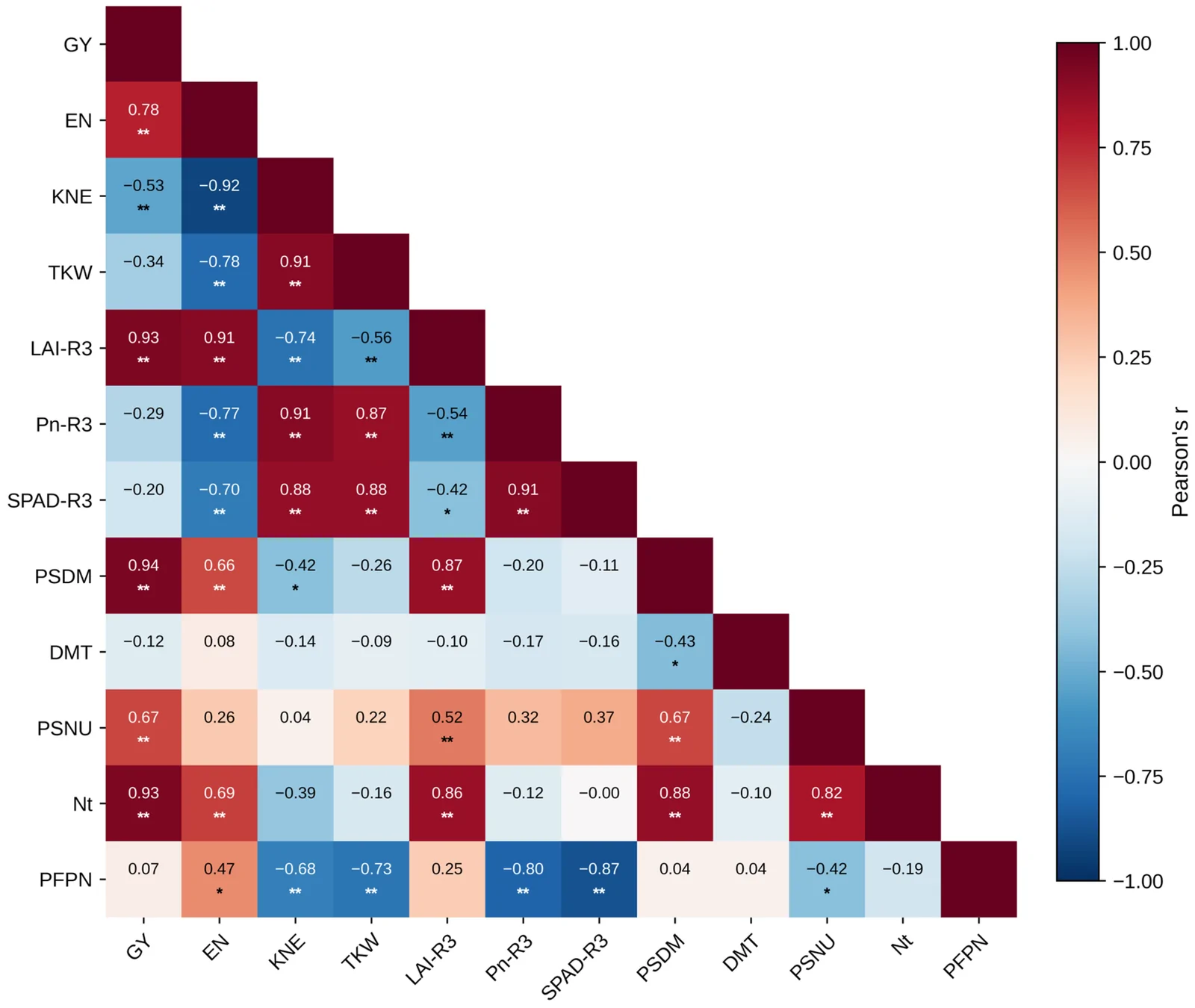
Pearson correlation coefficients among grain yield and yield components, canopy and leaf traits, and dry matter and nitrogen traits of spring maize (*n* = 24; 4 treatments × 3 replicates × 2 years). GY, grain yield; EN, ear number; KNE, kernels per ear; TKW, 1000-kernel weight; LAI-R3, leaf area index at the milk stage; *P*_n_-R3 and SPAD-R3, net photosynthetic rate and SPAD value of the ear leaf at the milk stage; PSDM, post-silking dry matter accumulation; DMT, pre-silking dry matter translocation; PSNU, post-silking N uptake; N_t_, total N accumulation at maturity; PFPN, partial factor productivity of N. * and ** indicate significance at *p* < 0.05 and *p* < 0.01, respectively. The correlations were calculated from plot-level data pooled across years and treatments and therefore largely reflect differences among treatment groups; they are presented as an exploratory analysis. Partial correlations after removing the effects of year and treatments are given in Table S3.

Across all plots (*n* = 24), grain yield was positively correlated with PSDM (*r* = 0.94), LAI at R3 (*r* = 0.93), N_t_ (*r* = 0.93), ear number (*r* = 0.78), and PSNU (*r* = 0.67) (all *p* < 0.01), and negatively correlated with kernels per ear (*r* = −0.53, *p* < 0.01) (Figure 4). Because these pooled correlations include the variation among years, densities, and N rates, they largely reflect differences among treatment groups rather than general physiological relationships; for example, the negative correlation between grain yield and kernels per ear arose because D2 increased yield while decreasing kernels per ear, and it should not be interpreted as a general biological relationship. Grain yield was not correlated with *P*_n_ at R3 across all plots (*r* = −0.29, *p* > 0.05), because D2 decreased leaf *P*_n_ while increasing canopy LAI. Within D2 (*n* = 12), however, grain yield was closely correlated with *P*_n_ (*r* = 0.91), SPAD value (*r* = 0.86), and LAI at R6 (*r* = 0.95), as well as with PSDM (*r* = 0.95) and PSNU (*r* = 0.87) (all *p* < 0.01). Within D1, only PSDM was significantly correlated with yield (*r* = 0.72, *p* < 0.01). Even these within-density correlations are still dominated by the contrast between the two N rates. To examine associations independent of treatment effects, partial correlations were calculated after removing the effects of year, density, N rate, and their interactions (Table S3). After this adjustment, grain yield remained positively associated with PSDM (*r* = 0.68, *p* < 0.01) and N_t_ (*r* = 0.57, *p* < 0.05), whereas its associations with LAI at R3, *P*_n_ and SPAD value at R3, and PSNU were weaker and not significant (|*r*| ≤ 0.45, *p* > 0.05), and the negative association with kernels per ear was no longer present (*r* = 0.22, *p* > 0.05). The correlation analysis is therefore regarded as exploratory and is used only to describe co-variation among traits, not to infer causal relationships.

**Figure 5:**
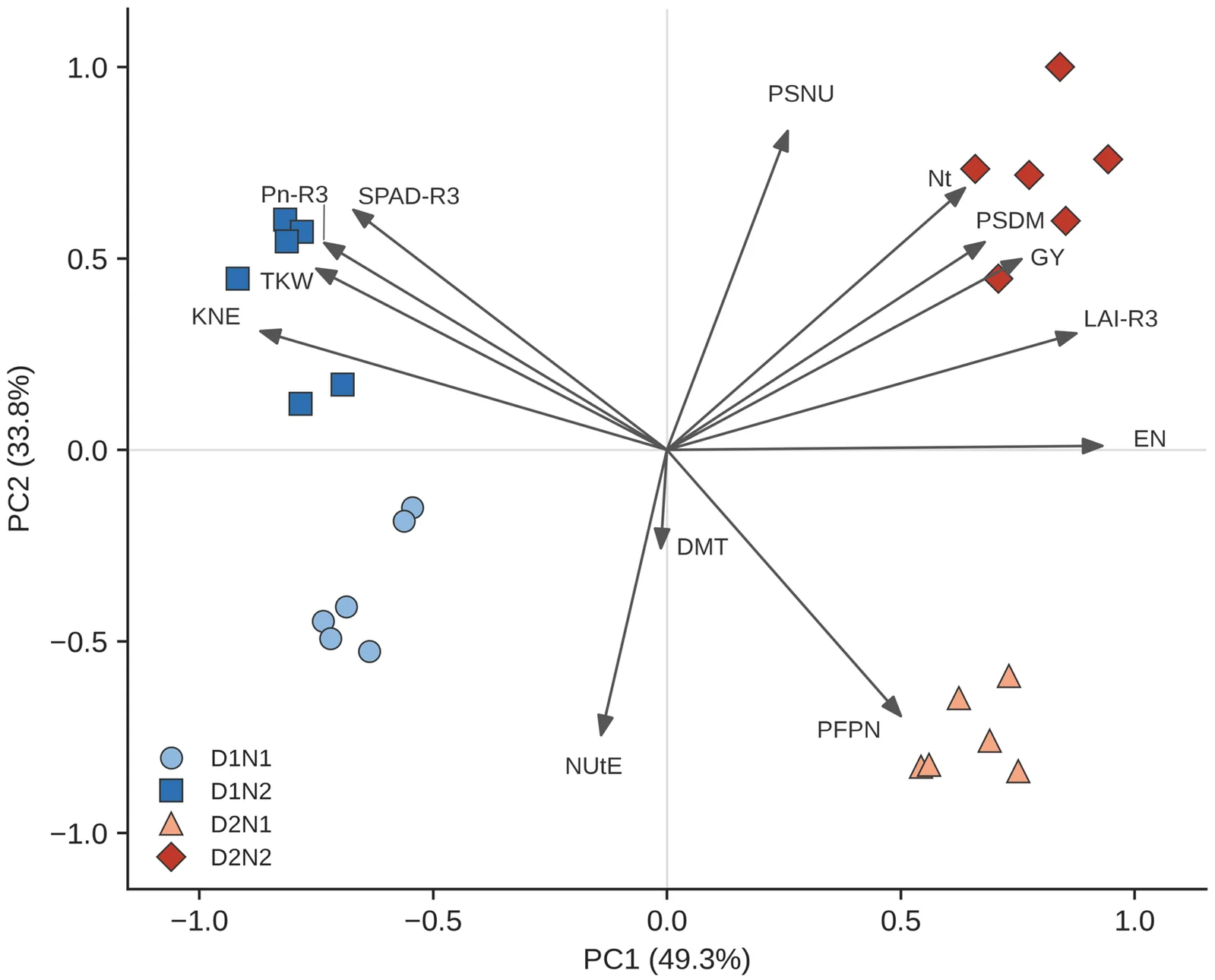
Principal component analysis biplot of yield, physiological, and N traits of spring maize under two planting densities and two N rates. Points are individual plots (*n* = 24); arrows are trait loadings. NUtE, N utilization efficiency; other abbreviations are as in Figure 4. The PCA is presented as an exploratory analysis.

As an exploratory analysis, principal component analysis (PCA) showed that the first two principal components (PCs) explained 49.3% and 33.8% of the total variation, respectively (Figure 5). PC1 separated the two densities: ear number (loading 0.98) and LAI at R3 (0.93) loaded positively, whereas kernels per ear (−0.92), 1000-kernel weight (−0.79), and *P*_n_ at R3 (−0.77) loaded negatively. PC2 separated the two N rates and was associated positively with PSNU (0.88), N_t_ (0.72), SPAD value (0.66), PSDM (0.58), and *P*_n_ (0.57), and negatively with NUtE (−0.79) and PFPN (−0.74). D2N2 was located in the quadrant associated with high grain yield, PSDM, and PSNU, whereas D2N1 was associated with high PFPN, and the two N1 treatments were located on the negative side of PC2, which was associated with high NUtE. Because the PCA was performed on plot-level data pooled across years and treatments, it mainly visualizes the separation among treatment groups and does not provide independent confirmation of the proposed physiological mechanisms.

## 3. Discussion

### 3.1. Dense Planting Increased Yield Mainly Through Ear Number at the Cost of Per-Plant Productivity

Increasing density from 60,000 to 90,000 plants ha^−1^ increased grain yield by 13.9%, which supports our first hypothesis. The yield gain resulted from a 46.8% increase in ear number, which more than compensated for the reductions in kernels per ear and 1000-kernel weight. This agrees with the view that yield gains of modern hybrids at high density come mainly from more ears per unit area rather than from larger ears [10,11,31]. The magnitude of the gain was also close to the 11.2% average yield increase in response to higher density reported in a global meta-analysis [13]. The decrease in kernels per ear under D2 is consistent with the reduction in per-plant growth rate around silking that determines kernel set [14,16], and the lower 1000-kernel weight reflects a smaller assimilate supply per kernel during grain filling [34,39]. The slight decrease in HI under D2 is also consistent with previous reports that crowding stress reduces HI [13,15].

The higher LAI under D2 increased canopy size and DM accumulation before silking, which agrees with the finding that dense planting improves light capture during the vegetative stage [12,13]. However, the same canopy also showed lower ear-leaf *P*_n_, *g*_s_, and SPAD values, especially at R3. One possible explanation is that individual leaves in dense canopies receive less light and have a lower N concentration (N dilution), which could hasten leaf senescence during grain filling [14,40]. However, canopy light distribution, leaf N concentration, and leaf senescence were not measured directly in this study, so this explanation remains a hypothesis that is consistent with, but not demonstrated by, the lower *P*_n_, SPAD value, and late-season LAI under D2. In terms of yield components, the per-plant yield penalty of dense planting appeared around silking, as fewer kernels per ear, and was followed after silking by a faster decline in green leaf area and lighter kernels. Under N1, D2 increased DM at R1 by 15.0% but PSDM by only 7.3%, consistent with the meta-analysis showing that density-driven yield gains rely mainly on pre-silking DM production [13]; under N2, however, D2 increased PSDM by 27.1%, suggesting that the higher N rate allowed dense stands to also benefit from post-silking production. This may help explain why the yield response to N differed between densities.

### 3.2. Enhanced N Supply Was Associated with Sustained Post-Silking Canopy Function Under Dense Planting

The significant D × N interaction showed that the benefit of additional N depended on density: N2 increased yield by 13.8% under D2 but only by 3.6% under D1. We hypothesize that, under low density, the soil N supply plus 180 kg N ha^−1^ was close to sufficient for plant demand, so that the additional N was largely not converted into yield; this inference is based only on the small, non-significant yield response and the moderate increase in plant N accumulation (+12.1% in N_t_) under D1. Under high density, by contrast, population N accumulation was larger (N_t_ of 233.0 vs. 194.3 kg ha^−1^ under N2), and the low PSNU of D2N1 is consistent with the hypothesis that N availability after silking limited D2N1, in line with the finding that crowding stress reduces the ability of maize to use soil N after silking [23,24]. Because soil mineral N, leaf N concentration, and fertilizer-N recovery were not measured during the season, neither the sufficiency of N supply under D1N1 nor post-silking N limitation under D2N1 can be confirmed; both interpretations should therefore be regarded as hypotheses inferred from grain yield and plant N accumulation.

Additional N acted both before and after silking, and these pre- and post-silking effects are likely to be interconnected rather than independent. Before silking, N2 increased N accumulation at R1 by a similar amount under both densities (16.6 and 17.9 kg ha^−1^ under D1 and D2, respectively) and had little effect on LAI at R1; under D2, however, it already increased the SPAD value at R1 (from 55.3 to 57.3) and kernels per ear (+9.1%), indicating that plant N status around silking was also improved. Because the increase in N accumulation at R1 was of similar magnitude at both densities, this pre-silking response alone is unlikely to account for the D × N interaction for grain yield. After silking, the responses diverged more strongly between densities: under D2, N2 reduced the post-silking LAI decline from 67.3% to 51.2%, increased *P*_n_ at R3 by 14.1%, and increased PSDM and PSNU by 22.0% and 44.3%, whereas under D1 it increased PSDM and PSNU by only 3.1% and 8.0%. These results partly support our second hypothesis: the density-dependent N response was most evident after silking, but it cannot be attributed to a single growth period, because the larger N pool and better N status at silking in D2N2 may have contributed both to kernel set and to the subsequent maintenance of green leaf area. The higher *C*_i_ combined with the lower *P*_n_ in D2N1 at R3 suggests that the lower photosynthetic rate was associated mainly with non-stomatal limitations, such as reduced leaf N and chlorophyll, rather than with stomatal closure. The lower SPAD value of D2N1 is consistent with this interpretation, although leaf N concentration was not measured. Because post-silking assimilates contributed 73.7–79.7% of grain DM in this study (two-year means; Table 3), maintaining leaf function during grain filling is likely to be an important process through which N alleviated the per-plant yield penalty of dense planting [19,20]. The slower LAI decline, together with the higher SPAD value and *P*_n_ at R3, is consistent with delayed leaf senescence under D2N2; however, senescence itself (e.g., the time course of green leaf number or leaf N concentration) was not measured, so delayed senescence remains an indirect inference. The strong correlations between grain yield and *P*_n_, SPAD value, and LAI at R6 within D2, but not within D1, largely reflect the N-rate contrast within D2 and are consistent with, rather than evidence for, a post-silking canopy limitation of yield in dense stands.

Changes in N dynamics are consistent with this interpretation. N accumulation at R1 and R6 was measured directly, whereas NT and PSNU are apparent values calculated from the differences in N accumulation between these two stages (Section 4.4.3) and do not trace the actual fluxes of remobilized or newly absorbed N. Under D2, N2 increased the apparent PSNU by 44.3%, whereas the apparent NT was similar between N rates (60.8 vs. 60.3 kg ha^−1^). Grain N is supplied by both remobilization of N stored before silking and N taken up after silking, and the balance between these sources affects leaf senescence [37,41]. When post-silking N uptake is limited, more N must be remobilized from leaves, which accelerates their senescence [40,42]. The higher PSNU in D2N2 may therefore have allowed grain N demand to be met with less depletion of leaf N, contributing to its slower LAI decline and higher SPAD value at R3, in line with reports that high-yielding modern hybrids accumulate more N after silking [36,43]. Although CNT was numerically higher in D2N1, the difference was not significant, and direct measurements of leaf N dynamics during grain filling are needed to confirm this mechanism. The 9.1% increase in kernels per ear under D2N2 likely reflects better plant N status during the critical period around silking, possibly including reduced kernel abortion early in grain filling [14,16], whereas the 4.6% increase in 1000-kernel weight is consistent with a sustained assimilate supply during grain filling, in agreement with the finding that higher N supply partly offsets the decrease in kernel weight under high density [34].

### 3.3. Balancing Grain Yield and Nitrogen Use Efficiency

D2N2 produced the highest yield, but D2N1 achieved the highest PFPN, and NUtE was decreased by N2 at both densities without differing significantly between D1N1 and D2N1, indicating a trade-off between maximizing yield and maximizing yield per unit of applied N. Dense planting improved PFPN at both N rates because it increased grain yield without additional N input, and the larger population also took up more N (Table 4), in agreement with previous studies showing that higher densities increase NUE at moderate to high N rates [23,30,31]. The marginal yield response to the additional 60 kg N ha^−1^ was about 23 kg grain per kg N under D2 but only about 6 kg under D1, so PFPN declined less with increasing N rate under D2 (14.7%) than under D1 (22.3%), indicating that the additional N gave a larger yield return in dense stands. However, PFPN is a partial productivity index (grain yield per unit of applied N) that does not separate the contribution of soil N from that of fertilizer N. Because a zero-N control was not included, agronomic N efficiency and apparent N recovery efficiency could not be calculated, and N losses (e.g., nitrate leaching and gaseous emissions) were not measured; therefore, the higher PFPN under D2 should not be interpreted as higher fertilizer-N recovery or lower environmental N losses.

Within the two N rates tested, the choice under the higher density depends on the production objective, and D2N2 and D2N1 should be regarded as options for different objectives rather than as experimentally validated economic or environmental optima, because fertilizer costs, economic returns, and N losses were not evaluated. Where maximum grain yield is the objective, D2N2 increased yield by 1.40 t ha^−1^ over D2N1 with an extra 60 kg N ha^−1^. Where reducing N input is the objective, D2N1 still yielded 4.7% and 8.5% more than D1N2 and D1N1, respectively, with the highest PFPN. Which option is economically or environmentally preferable will depend on grain and fertilizer prices and on N losses, which should be quantified in future studies. For drip-fertigated spring maize in northwest China, Lai et al. [30] found that 95,000–110,000 plants ha^−1^ combined with 185–205 kg N ha^−1^ achieved at least 95% of the maximum grain yield, economic benefit, and water productivity, which is close to the density and N range tested in the present study. By contrast, drip-irrigated maize managed for yields above 17 t ha^−1^ at 105,000 plants ha^−1^ or more in Xinjiang required about 430–470 kg N ha^−1^ to reach the optimum yield [29], and in high-yield fields at Qitai, matching the vertical distributions of canopy N and light was a key trait for yield improvement once N demand was met at 360 kg N ha^−1^ [33]. These contrasts suggest that the N requirement of dense maize in this region increases with the target yield and density, so N recommendations for dense planting should be based on the yield goal and site-specific soil N supply [4,28].

### 3.4. Limitations and Future Perspectives

This study was conducted at one site with one hybrid over two growing seasons, and only two levels of each factor were tested. Therefore, the optimal density and N rate could not be determined from response curves. In particular, the higher density tested (90,000 plants ha^−1^) is below the densities of 120,000–135,000 plants ha^−1^ at which the highest yields have been obtained in Xinjiang [5,6], so the N response of maize at these densities remains to be established. In addition, the absence of a zero-N control did not allow the calculation of agronomic N efficiency and apparent N recovery efficiency, and N losses to the environment, such as nitrate leaching below the root zone, were not measured. Leaf N concentration during grain filling and soil mineral N during the season were also not monitored, so the roles of N remobilization and soil N supply were inferred indirectly. Likewise, canopy light distribution and the time course of leaf senescence were not measured, so the roles of shading, N dilution, and delayed senescence were inferred from LAI, SPAD, and gas-exchange data, and the correlation analysis and PCA were exploratory. Fertilizer costs and economic returns were also not evaluated. Irrigation was applied uniformly to all treatments, so possible interactions among water supply, density, and N [8,29] were not examined. Lodging did not occur during the two seasons, but the risk of lodging at high density under strong winds should be considered [18]. Future studies should include more density and N levels, a zero-N control, contrasting hybrids, and multiple sites, and should examine root distribution, N losses, and the timing of N fertigation to further optimize N management for dense planting.

## 4. Materials and Methods

### 4.1. Experimental Site

Field experiments were conducted during the 2024 and 2025 maize growing seasons at the Water-Saving Irrigation Experimental Station of Shihezi University, Shihezi, Xinjiang, China (44°19′ N, 85°59′ E; altitude 451 m). The site lies in an oasis on the northern slope of the Tianshan Mountains and has a temperate continental arid climate, with a long-term mean annual temperature of about 7.7 °C, annual precipitation of about 207 mm, annual evaporation of about 1660 mm, annual sunshine duration of about 2865 h, accumulated temperature above 10 °C of about 3460 °C, and a frost-free period of about 170 d [7]. Because growing-season precipitation is far below crop water demand, maize production depends on irrigation [38]. Air temperature and precipitation during the two growing seasons were recorded by an automatic weather station (TRM-ZS2, Jinzhou Sunshine Meteorological Technology Co., Ltd., Jinzhou, China) located at the experimental site. During the maize growing season (April–September), the mean air temperature was 21.3 °C in 2024 and 21.7 °C in 2025, and total precipitation was 132.4 and 109.7 mm, respectively, compared with long-term means of 20.8 °C and 124.9 mm for the same period (Figure S1 and Table S1). Both seasons were thus slightly warmer than the long-term average, and growing-season precipitation accounted for less than one-quarter of the total water input (precipitation plus 480 mm of irrigation). The soil is a grey desert soil with a medium loam texture, and the properties of the 0–20 cm soil layer before sowing are given in Table 5. The previous crop was maize in both years, and the groundwater table is deeper than 10 m.

### 4.2. Plant Material and Experimental Design

The maize hybrid ‘Xianyu 335’, which is widely grown in northern Xinjiang, was used in both years; seeds were obtained from Tieling Pioneer Seed Research Co., Ltd., Tieling, China. The experiment was arranged in a split-plot design with three replicates. Main plots were two planting densities, 60,000 plants ha^−1^ (D1, low density) and 90,000 plants ha^−1^ (D2, high density). Subplots were two N rates, 180 kg N ha^−1^ (N1) and 240 kg N ha^−1^ (N2). In each year, the three replicates were arranged as complete blocks; density main plots were randomized within each block, and N subplots were randomized within each main plot. The experiment was conducted in the same field in both years, and each treatment was kept in the same plot in both years. Each subplot was 10 m long and 4.8 m wide (eight rows with 60 cm row spacing), and plant spacing within rows was 27.8 cm for D1 and 18.5 cm for D2. Plastic film (0.01 mm thick) and drip tapes (Xinjiang Tianye Water Saving Irrigation System Co., Ltd., Shihezi, China) were laid at sowing with a combined seeder; each film strip covered two rows, with one drip tape placed between the two rows (emitter spacing 30 cm; emitter discharge 2.6 L h^−1^). Maize was sown on 20 April 2024 and 22 April 2025 with two seeds per hill and thinned to the target density at the three-leaf stage. Plants reached physiological maturity in mid-September and were harvested on 18 September 2024 and 20 September 2025.

### 4.3. Field Management

Irrigation was applied through the drip system 10 times during the growing season at intervals of 7–10 d from V6 (early June) to R5 (late August), with a total amount of 450 mm in each year, following local practice for drip-irrigated maize in northern Xinjiang [38]. An additional 30 mm was applied immediately after sowing to ensure uniform emergence. The irrigation volume of each plot was controlled with a water meter. N was applied as urea (46% N): 20% of the N was applied as basal fertilizer before sowing, and the remaining 80% was applied by fertigation in 6 doses from V6 to R3 using a pressure-differential fertilizer tank (Xinjiang Tianye Water Saving Irrigation System Co., Ltd.) connected to each subplot. The six fertigation events were applied at V6, V9, V12, VT, R2, and R3 and supplied 10%, 15%, 15%, 15%, 15%, and 10% of the total N rate, respectively (i.e., 25% of the N was applied after silking), and the same schedule was used in both years. All plots received 90 kg P_2_O_5_ ha^−1^ as triple superphosphate and 90 kg K_2_O ha^−1^ as potassium sulfate before sowing. All fertilizers were purchased from a local agricultural supply store in Shihezi, China. Weeds, insect pests, and diseases were controlled according to local practice, and no lodging, obvious water stress, or disease damage occurred during the experiment.

### 4.4. Sampling and Measurements

#### 4.4.1. Leaf Area Index

Growth stages were determined according to Ritchie et al. [45]. At V6, V12, R1, R3, and R6 (about 42, 60, 85, 105, and 145 days after sowing, respectively), three representative plants were sampled from the inner rows of each subplot. The same plants were subsequently used to determine DM and N accumulation (Section 4.4.3). The length (L) and maximum width (W) of all green leaves were measured, and LAI was calculated as follows [46]:

(1) $$LAI=\frac{\sum_{i=1}^{n}\left(L_{i}\times W_{i}\times 0.75\right)\times \rho }{10,000}$$

where *L*_i_ and *W*_i_ (cm) are the length and maximum width of leaf *i*, 0.75 is the leaf shape coefficient, *n* is the number of green leaves per plant, and *ρ* is the planting density (plants m^−2^). The LAI values of the three plants were averaged to obtain one value per subplot.

#### 4.4.2. Photosynthetic Parameters and SPAD Value

At R1 and R3, *P*_n_, *g*_s_, and *C*_i_ of the middle part of the ear leaf were measured on five plants per subplot between 11:00 and 13:30 Beijing time (about 2 h ahead of local solar time) on clear days using a portable photosynthesis system (LI-6800, LI-COR, Lincoln, NE, USA). The photosynthetic photon flux density was set at 1800 µmol m^−2^ s^−1^, the reference CO_2_ concentration at 400 µmol mol^−1^, and the leaf temperature at 30 °C. The SPAD value of the same leaves was measured using a chlorophyll meter (SPAD-502Plus, Konica Minolta, Tokyo, Japan), and the mean of five readings per leaf was recorded. The values of the five plants were averaged to obtain one value per subplot before statistical analysis.

#### 4.4.3. Dry Matter and N Accumulation and Translocation

Sampled plants were separated into leaves, stems (including sheaths and tassels), and ears at R1, and into leaves, stems, cobs plus husks, and grain at R6. Samples were oven-dried at 105 °C for 30 min and then at 75 °C to constant weight, weighed, and ground. N concentration was determined by the Kjeldahl method after digestion with H_2_SO_4_ and H_2_O_2_ (Xinjiang Takelan Biotechnology Co., Ltd., Shihezi, China) [44], and N accumulation was calculated as DM multiplied by N concentration. Per-plant values were averaged over the three sampled plants. DM translocation parameters were calculated as follows [20,31]:

(2) $$DMT\;\left(t\;{ha}^{-1}\right)={DM}_{veg,R1}-{DM}_{veg,R6}$$

(3) $$DMTE\;\left(\%\right)=\frac{DMT}{{DM}_{veg,R1}}\times 100$$

(4) $$CDMT\;\left(\%\right)=\frac{DMT}{GDM}\times 100$$

(5) $$PSDM\;\left(t\;{ha}^{-1}\right)={DM}_{R6}-{DM}_{R1}$$

(6) $$CPSA\;\left(\%\right)=\frac{GDM-DMT}{GDM}\times 100$$

where DM_veg,R1_ and DM_veg,R6_ are the DM of vegetative organs (leaves and stems) at R1 and R6, DM_R1_ and DM_R6_ are the total aboveground DM at R1 and R6, and GDM is the grain DM at R6. DMTE is the DM translocation efficiency. Pre-silking N translocation (NT), its contribution to grain N (CNT), and post-silking N uptake (PSNU) were calculated in the same way from N accumulation:

(7) $$NT\;\left(kg\;{ha}^{-1}\right)=N_{veg,R1}-N_{veg,R6}$$

(8) $$CNT\;\left(\%\right)=\frac{NT}{N_{grain}}\times 100$$

(9) $$PSNU\;\left(kg\;{ha}^{-1}\right)=N_{R6}-N_{R1}$$

where *N*_veg,R1_ and *N*_veg,R6_ are the N accumulation in vegetative organs (leaves and stems) at R1 and R6, *N*_R1_ and *N*_R6_ are the total aboveground N accumulation at R1 and R6, and *N*_grain_ is the grain N accumulation at R6. These indices are apparent values based on the assumptions that (i) the decrease in vegetative N between R1 and R6 was entirely translocated to the grain, i.e., N losses from vegetative organs (e.g., through leaves shed before sampling or gaseous losses from the canopy) were negligible, and (ii) the net change in aboveground N between R1 and R6 represents post-silking N uptake. Therefore, NT, CNT, and PSNU describe the apparent N balance between R1 and R6 rather than directly measured remobilization or uptake fluxes, which would require ^15^N labelling. Senesced leaves still attached to the plants at R6 were included in the leaf fraction. CPSA is the contribution of post-silking assimilates to grain DM. Because PSDM also includes the post-silking growth of cobs and husks, the ratio PSDM/GDM overestimates the contribution of post-silking assimilation to grain; CPSA was therefore calculated as the complement of CDMT, so that CDMT + CPSA = 100%.

#### 4.4.4. Determination of Grain Yield and Yield Components

At R6, all ears in the central four rows over 5 m of each subplot (12 m^2^) were harvested, and the number of ears was counted. Twenty consecutive ears were used to determine the number of kernels per ear and the 1000-kernel weight. Grain moisture was measured with a grain moisture meter (PM-8188-A, Kett Electric Laboratory, Tokyo, Japan), and grain yield was adjusted to 14% moisture content:

(10) $$GY\;\left(t\;{ha}^{-1}\right)=\frac{W\times \left(100-M\right)}{100-14}\times \frac{10}{A}$$

where *W* is the fresh grain weight (kg), *M* is the grain moisture content (%), and *A* is the harvested area (m^2^). HI was calculated as GDM divided by the total aboveground DM at R6.

#### 4.4.5. Nitrogen Use Efficiency

NUE indices were calculated as follows [28,47,48]:

(11) $$PFPN\;\left(kg\;{kg}^{-1}\right)=\frac{1000\times GY}{N_{rate}}$$

(12) $$NUtE\;\left(kg\;{kg}^{-1}\right)=\frac{1000\times GY}{N_{t}}$$

(13) $$NHI\;\left(\%\right)=\frac{N_{grain}}{N_{t}}\times 100$$

where GY is the grain yield (t ha^−1^) from Equation (10), multiplied by 1000 to express it in kg ha^−1^, *N*_rate_ is the N application rate (kg ha^−1^), *N*_t_ is the total aboveground N accumulation at R6 (kg ha^−1^), and *N*_grain_ is the grain N accumulation at R6 (kg ha^−1^).

### 4.5. Statistical Analysis

The subplot was the experimental unit. Measurements on individual plants within a subplot (three plants for LAI, DM, and N; five plants for gas exchange and SPAD value) were treated as subsamples and averaged to one value per subplot before analysis, giving three replicates per treatment in each year and 24 observations in total. Normality of the residuals was checked with the Shapiro–Wilk test and homogeneity of variance with Levene’s test, and no data transformation was required. A combined split-plot analysis of variance across the two years was performed with year, density, and N rate as fixed factors and replicate nested within year as a random factor. The effect of year was tested against the replicate-within-year mean square, the effects of density and year × density were tested against the main-plot error (replicate × density within year), and all other effects were tested against the subplot error. The corresponding error degrees of freedom were 4 (replicate within year), 4 (main-plot error), and 8 (subplot error). Because neither year nor any interaction involving year was significant for the main traits (Table 1), inference on the main effects and the D × N interaction was based on the combined analysis. To show the consistency of treatment responses between years, the means of the four treatment combinations were also compared within each year using the least significant difference (LSD) test at *p* < 0.05; these year-specific groupings are descriptive and are interpreted together with the combined analysis. For the ear-leaf traits in Figure 2, which are presented as two-year means (*n* = 6), treatment means were compared using the LSD test. Percentage changes given in the text were calculated from unrounded treatment means and may therefore differ by up to 0.1 percentage point from values recalculated from the rounded means shown in the tables and figures. Pearson correlation analysis and principal component analysis (PCA; on standardized data) were performed on plot-level values (*n* = 24) pooled across years and treatments to explore the relationships between grain yield and physiological traits. Because these analyses do not account for the nested structure of years and replicates or for treatment effects, they were regarded as exploratory. To assess whether the associations persisted after accounting for treatment effects, partial correlation coefficients were also calculated after removing the effects of year, density, N rate, and all their interactions (i.e., correlations among the residuals of the three-way factorial model; *df* = 15). Statistical analyses were performed with SPSS 26.0 (IBM Corp., Armonk, NY, USA), and figures were prepared with Origin 2024 (OriginLab Corp., Northampton, MA, USA) and Python 3.11 (Python Software Foundation, Wilmington, DE, USA) with the matplotlib and seaborn libraries.

## 5. Conclusions

Increasing planting density from 60,000 to 90,000 plants ha^−1^ increased the grain yield of drip-irrigated spring maize by 13.9% in a two-year field experiment with one hybrid at Shihezi, Xinjiang, mainly by increasing ear number. The response to N rate depended on density: increasing the N rate from 180 to 240 kg N ha^−1^ increased yield by 13.8% under high density but had no significant effect under low density. The higher N rate increased N accumulation before silking to a similar extent at both densities and, under high density, also improved plant N status around silking and kernels per ear. Its additional benefit under high density was most evident after silking, when it slowed the decline in green leaf area, maintained a higher ear-leaf SPAD value and photosynthetic rate, and increased post-silking DM accumulation and apparent N uptake; these responses are consistent with, but do not directly demonstrate, delayed leaf senescence. Within the two N rates tested, D2N2 (240 kg N ha^−1^) achieved the highest yield (11.51 t ha^−1^), whereas D2N1 (180 kg N ha^−1^) achieved the highest PFPN (56.2 kg kg^−1^). These two combinations represent options for yield-oriented and N-input-saving objectives, respectively, rather than economic or environmental optima, because fertilizer costs, economic returns, and N losses were not assessed. Studies across multiple sites, hybrids, and a wider range of planting densities (e.g., 105,000 plants ha^−1^ or more) and N rates, including a zero-N control, are needed before these results can be generalized to other arid oasis regions.

## Figures and Tables

**Table 1:** *F* values from the split-plot analysis of variance for (**a**) grain yield, yield components, and dry matter and nitrogen (N) traits and (**b**) leaf area index (LAI) and ear-leaf photosynthetic traits of spring maize in 2024 and 2025.

(a)									
Source of Variation	df	GY	EN	KNE	TKW	HI	PSDM	N_t_	PFPN
Year (Y)	1	5.55 ns	0.04 ns	0.01 ns	0.20 ns	3.08 ns	4.84 ns	0.85 ns	4.95 ns
Density (D)	1	206.76 **	1567.34 **	440.41 **	681.20 **	14.56 *	79.93 **	769.64 **	232.06 **
Nitrogen (N)	1	102.25 **	2.72 ns	122.12 **	40.82 **	1.00 ns	20.69 **	331.82 **	541.44 **
Y × D	1	0.04 ns	2.38 ns	0.22 ns	2.50 ns	0.28 ns	0.37 ns	0.67 ns	0.09 ns
Y × N	1	0.21 ns	0.01 ns	0.05 ns	2.96 ns	2.63 ns	0.39 ns	0.13 ns	0.80 ns
D × N	1	38.22 **	0.00 ns	20.07 **	1.21 ns	1.47 ns	12.21 **	32.84 **	15.16 **
Y × D × N	1	0.35 ns	2.39 ns	1.35 ns	0.06 ns	0.00 ns	1.32 ns	0.05 ns	0.37 ns
**(b)**									
**Source of variation**	**df**	**LAI-R1**	**LAI-R6**	***P*** **_n_** **-R1**	***P*** **_n_** **-R3**	***g*** **_s_** **-R1**	***g*** **_s_** **-R3**	**SPAD-R1**	**SPAD-R3**
Year (Y)	1	1.12 ns	0.37 ns	2.84 ns	3.65 ns	1.95 ns	2.17 ns	0.92 ns	1.48 ns
Density (D)	1	486.35 **	58.62 **	96.17 **	312.74 **	64.28 **	205.36 **	41.53 **	176.92 **
Nitrogen (N)	1	4.37 ns	152.48 **	9.86 *	128.53 **	7.41 *	87.94 **	13.06 **	214.37 **
Y × D	1	0.52 ns	0.09 ns	0.31 ns	0.85 ns	0.22 ns	0.64 ns	0.47 ns	0.27 ns
Y × N	1	0.08 ns	0.71 ns	0.47 ns	0.19 ns	0.35 ns	0.12 ns	0.26 ns	0.58 ns
D × N	1	0.64 ns	37.04 **	4.12 ns	12.36 **	0.03 ns	26.51 **	5.81 *	16.66 **
Y × D × N	1	0.11 ns	0.24 ns	0.06 ns	0.43 ns	0.18 ns	0.29 ns	0.33 ns	0.15 ns

GY, grain yield; EN, ear number; KNE, kernels per ear; TKW, 1000-kernel weight; HI, harvest index; PSDM, post-silking dry matter accumulation; N_t_, total N accumulation at maturity; PFPN, partial factor productivity of N. LAI-R1 and LAI-R6, leaf area index at silking (R1) and physiological maturity (R6); *P*_n_, *g*_s_, and SPAD, net photosynthetic rate, stomatal conductance, and SPAD value of the ear leaf at R1 and the milk stage (R3). Year was tested against replicate (year); D and Y × D against the main-plot error; other effects against the subplot error. The error degrees of freedom were 4 for replicate (year), 4 for the main-plot error, and 8 for the subplot error. * and ** indicate significance at *p* < 0.05 and *p* < 0.01, respectively; ns, not significant.

**Table 2:** Grain yield and yield components of spring maize under two planting densities and two N rates in 2024 and 2025.

Year	Treatment	EN (×10^4^ ha^−1^)	KNE	TKW (g)	GY (t ha^−1^)	HI
2024	D1N1	5.78 b	530 b	324.7 a	9.20 c	0.494 a
	D1N2	6.00 b	550 a	330.7 a	9.53 bc	0.497 a
	D2N1	8.51 a	431 d	299.0 c	9.93 b	0.478 b
	D2N2	8.52 a	468 c	310.0 b	11.41 a	0.489 ab
2025	D1N1	5.79 b	536 a	321.7 b	9.44 c	0.500 a
	D1N2	5.79 b	549 a	335.0 a	9.79 c	0.495 a
	D2N1	8.53 a	427 c	299.6 c	10.30 b	0.487 a
	D2N2	8.73 a	468 b	316.0 b	11.61 a	0.489 a

D1 and D2, 60,000 and 90,000 plants ha^−1^; N1 and N2, 180 and 240 kg N ha^−1^. EN, ear number; KNE, kernels per ear; TKW, 1000-kernel weight; GY, grain yield at 14% moisture; HI, harvest index. Values are means (*n* = 3). Different lowercase letters within a column in the same year indicate significant differences (LSD, *p* < 0.05).

**Table 3:** Dry matter (DM) accumulation at silking and maturity, pre-silking DM translocation, and post-silking DM accumulation of spring maize in 2024 and 2025.

Year	Treatment	DM at R1 (t ha^−1^)	DM at R6 (t ha^−1^)	DMT (t ha^−1^)	DMTE (%)	CDMT (%)	PSDM (t ha^−1^)	CPSA (%)
2024	D1N1	8.98 c	16.03 c	1.83 a	22.1 a	23.2 a	7.05 b	76.8 a
	D1N2	9.57 b	16.47 c	2.31 a	26.1 a	28.2 a	6.90 b	71.8 a
	D2N1	10.62 a	17.86 b	2.30 a	23.6 a	27.0 a	7.24 b	73.0 a
	D2N2	11.09 a	20.09 a	2.06 a	20.1 a	21.0 a	9.00 a	79.0 a
2025	D1N1	9.33 c	16.25 d	2.18 a	25.4 a	26.9 a	6.92 c	73.1 b
	D1N2	9.51 c	17.01 c	2.06 a	23.5 ab	24.5 ab	7.50 bc	75.5 ab
	D2N1	10.43 b	18.18 b	2.27 a	23.5 ab	25.6 ab	7.75 b	74.4 ab
	D2N2	11.13 a	20.42 a	1.96 a	19.2 b	19.7 b	9.30 a	80.3 a

R1, silking; R6, physiological maturity; DMT, pre-silking DM translocation; DMTE, DM translocation efficiency; CDMT, contribution of DMT to grain DM; PSDM, post-silking DM accumulation; CPSA, contribution of post-silking assimilates to grain DM. Treatment abbreviations are as in Table 2. Different lowercase letters within a column in the same year indicate significant differences (LSD, *p* < 0.05).

**Table 4:** Nitrogen (N) accumulation, translocation, and N use efficiency of spring maize in 2024 and 2025.

Year	Treatment	N at R1 (kg ha^−1^)	N_t_ (kg ha^−1^)	NT (kg ha^−1^)	CNT (%)	PSNU (kg ha^−1^)	NHI (%)	NUtE (kg kg^−1^)	PFPN (kg kg^−1^)
2024	D1N1	118.9 d	171.8 c	51.0 a	49.4 a	52.9 bc	60.2 a	53.5 a	51.1 b
	D1N2	134.0 c	193.7 b	51.3 a	46.1 a	59.7 b	57.4 a	49.2 b	39.7 d
	D2N1	140.6 b	190.8 b	57.5 a	53.1 a	50.2 c	56.7 a	52.0 ab	55.1 a
	D2N2	158.5 a	231.2 a	60.5 a	45.2 a	72.7 a	57.8 a	49.4 b	47.5 c
2025	D1N1	117.9 d	174.9 c	49.1 b	46.7 a	57.0 b	60.2 a	54.0 a	52.5 b
	D1N2	135.9 c	194.9 b	57.3 ab	49.5 a	59.0 b	59.4 ab	50.3 b	40.8 d
	D2N1	144.5 b	194.9 b	64.1 a	56.0 a	50.4 b	58.7 ab	52.9 ab	57.2 a
	D2N2	162.4 a	234.8 a	60.0 ab	45.3 a	72.4 a	56.4 b	49.5 b	48.4 c

N_t_, total N accumulation at maturity; NT, pre-silking N translocation; CNT, contribution of NT to grain N; PSNU, post-silking N uptake; NHI, N harvest index; NUtE, N utilization efficiency; PFPN, partial factor productivity of N. Treatment abbreviations are as in Table 2. Different lowercase letters within a column in the same year indicate significant differences (LSD, *p* < 0.05).

**Table 5:** Physicochemical properties of the topsoil (0–20 cm) at the experimental site before sowing.

Year	pH	Organic Matter (g kg^−1^)	Total N (g kg^−1^)	Alkali-Hydrolyzable N (mg kg^−1^)	Olsen-P (mg kg^−1^)	Available K (mg kg^−1^)
2024	8.12	14.6	0.92	68.5	21.3	118.6
2025	8.08	15.1	0.95	71.2	22.8	123.4

pH was measured in a 1:2.5 soil:water suspension; organic matter by potassium dichromate oxidation; total N by the Kjeldahl method; alkali-hydrolyzable N by alkaline diffusion; Olsen-P by NaHCO_3_ extraction; available K by NH_4_OAc extraction and flame photometry [44]. The reagents were purchased from Xinjiang Takelan Biotechnology Co., Ltd., Shihezi, China.

## Data Availability

The original contributions presented in this study are included in the article and its Supplementary Materials (Figure S1 and Tables S1–S3). Other data supporting the findings of this study, including the raw plot-level measurements, are not publicly available because they are part of an ongoing research project, but are available from the corresponding authors upon reasonable request.

## Supplementary Materials

The following supporting information can be downloaded at: https://www.mdpi.com/article/10.3390/plants15213467/s1, Figure S1: Monthly mean air temperature and monthly precipitation from April to September in 2024 and 2025 and the long-term means at the experimental site; Table S1: Monthly mean air temperature and precipitation during the maize growing seasons of 2024 and 2025 and the long-term means at the experimental site; Table S2: F values from the split-plot analysis of variance for leaf area index (LAI) at the sixth-leaf (V6), twelfth-leaf (V12), and milk (R3) stages and for the intercellular CO_2_ concentration (*C*_i_) of the ear leaf at silking (R1) and R3 in 2024 and 2025; Table S3: Pearson correlation coefficients calculated from pooled plot-level data and partial correlation coefficients after removing the effects of year, density, N rate, and their interactions, between grain yield and yield components, canopy and leaf traits, and dry matter and N traits.

## Abbreviations

The following abbreviations are used in this manuscript:

CDMT	Contribution of pre-silking DM translocation to grain DM
*C*_i_	Intercellular CO_2_ concentration
CNT	Contribution of pre-silking N translocation to grain N
CPSA	Contribution of post-silking assimilates to grain DM
D	Planting density
D1, D2	60,000 and 90,000 plants ha^−1^
DM	Dry matter
DMT	Pre-silking DM translocation
DMTE	DM translocation efficiency
EN	Ear number
GDM	Grain dry matter
*g*_s_	Stomatal conductance
GY	Grain yield
HI	Harvest index
KNE	Kernels per ear
LAI	Leaf area index
LSD	Least significant difference
N	Nitrogen
N1, N2	180 and 240 kg N ha^−1^
NHI	N harvest index
NT	Pre-silking N translocation
Nt	Total N accumulation at maturity
NUE	N use efficiency
NUtE	N utilization efficiency
PC	Principal component
PCA	Principal component analysis
PFPN	Partial factor productivity of N
*P*_n_	Net photosynthetic rate
PSDM	Post-silking DM accumulation
PSNU	Post-silking N uptake
R1, R2, R3, R5, R6	Silking, blister, milk, dent, and physiological maturity stages
SPAD	Leaf greenness (relative chlorophyll content) measured with a SPAD meter
TKW	1000-kernel weight
V6, V9, V12	Sixth-, ninth-, and twelfth-leaf stages
VT	Tasseling
Y	Year

## References

[1] Erenstein O., Jaleta M., Sonder K., Mottaleb K., Prasanna B.M. (2022). Global maize production, consumption and trade: Trends and R&D implications. Food Secur..

[2] Ranum P., Peña-Rosas J.P., Garcia-Casal M.N. (2014). Global maize production, utilization, and consumption. Ann. N. Y. Acad. Sci..

[3] Meng Q., Hou P., Wu L., Chen X., Cui Z., Zhang F. (2013). Understanding production potentials and yield gaps in intensive maize production in China. Field Crops Res..

[4] Chen X.-P., Cui Z.-L., Vitousek P.M., Cassman K.G., Matson P.A., Bai J.-S., Meng Q.-F., Hou P., Yue S.-C., Römheld V. (2011). Integrated soil–crop system management for food security. Proc. Natl. Acad. Sci. USA.

[5] Liu G., Hou P., Xie R., Ming B., Wang K., Xu W., Liu W., Yang Y., Li S. (2017). Canopy characteristics of high-yield maize with yield potential of 22.5 Mg ha^−1^. Field Crops Res..

[6] Zhang G., Ming B., Shen D., Xie R., Hou P., Xue J., Wang K., Li S. (2021). Optimizing grain yield and water use efficiency based on the relationship between leaf area index and evapotranspiration. Agriculture.

[7] Wang F., Wang Z., Zhang J., Li W. (2019). Combined effect of different amounts of irrigation and mulch films on physiological indexes and yield of drip-irrigated maize (*Zea mays* L.). Water.

[8] Wang F., Xiao J., Ming B., Xie R., Wang K., Hou P., Liu G., Zhang G., Chen J., Liu W. (2021). Grain yields and evapotranspiration dynamics of drip-irrigated maize under high plant density across arid to semi-humid climates. Agric. Water Manag..

[9] Duvick D.N. (2005). The contribution of breeding to yield advances in maize (*Zea mays* L.). Adv. Agron..

[10] Tollenaar M., Lee E.A. (2002). Yield potential, yield stability and stress tolerance in maize. Field Crops Res..

[11] Assefa Y., Vara Prasad P.V., Carter P., Hinds M., Bhalla G., Schon R., Jeschke M., Paszkiewicz S., Ciampitti I.A. (2016). Yield responses to planting density for US modern corn hybrids: A synthesis-analysis. Crop Sci..

[12] Hammer G.L., Dong Z., McLean G., Doherty A., Messina C., Schussler J., Zinselmeier C., Paszkiewicz S., Cooper M. (2009). Can changes in canopy and/or root system architecture explain historical maize yield trends in the U.S. Corn Belt?. Crop Sci..

[13] Shao H., Wu X., Duan J., Zhu F., Chi H., Liu J., Shi W., Xu Y., Wei Z., Mi G. (2024). How does increasing planting density regulate biomass production, allocation, and remobilization of maize temporally and spatially: A global meta-analysis. Field Crops Res..

[14] Boomsma C.R., Santini J.B., Tollenaar M., Vyn T.J. (2009). Maize morphophysiological responses to intense crowding and low nitrogen availability: An analysis and review. Agron. J..

[15] Tollenaar M., Deen W., Echarte L., Liu W. (2006). Effect of crowding stress on dry matter accumulation and harvest index in maize. Agron. J..

[16] Andrade F.H., Vega C., Uhart S., Cirilo A., Cantarero M., Valentinuz O. (1999). Kernel number determination in maize. Crop Sci..

[17] Rossini M.A., Maddonni G.A., Otegui M.E. (2011). Inter-plant competition for resources in maize crops grown under contrasting nitrogen supply and density: Variability in plant and ear growth. Field Crops Res..

[18] Li B., Gao F., Ren B., Dong S., Liu P., Zhao B., Zhang J. (2021). Lignin metabolism regulates lodging resistance of maize hybrids under varying planting density. J. Integr. Agric..

[19] Lee E.A., Tollenaar M. (2007). Physiological basis of successful breeding strategies for maize grain yield. Crop Sci..

[20] Ning P., Li S., Yu P., Zhang Y., Li C. (2013). Post-silking accumulation and partitioning of dry matter, nitrogen, phosphorus and potassium in maize varieties differing in leaf longevity. Field Crops Res..

[21] Ciampitti I.A., Vyn T.J. (2012). Physiological perspectives of changes over time in maize yield dependency on nitrogen uptake and associated nitrogen efficiencies: A review. Field Crops Res..

[22] Uhart S.A., Andrade F.H. (1995). Nitrogen deficiency in maize: I. Effects on crop growth, development, dry matter partitioning, and kernel set. Crop Sci..

[23] Ciampitti I.A., Vyn T.J. (2011). A comprehensive study of plant density consequences on nitrogen uptake dynamics of maize plants from vegetative to reproductive stages. Field Crops Res..

[24] Yan P., Pan J., Zhang W., Shi J., Chen X., Cui Z. (2017). A high plant density reduces the ability of maize to use soil nitrogen. PLoS ONE.

[25] Ju X.T., Xing G.X., Chen X.P., Zhang S.L., Zhang L.J., Liu X.J., Cui Z.L., Yin B., Christie P., Zhu Z.L. (2009). Reducing environmental risk by improving N management in intensive Chinese agricultural systems. Proc. Natl. Acad. Sci. USA.

[26] Zhang X., Davidson E.A., Mauzerall D.L., Searchinger T.D., Dumas P., Shen Y. (2015). Managing nitrogen for sustainable development. Nature.

[27] Chen X., Cui Z., Fan M., Vitousek P., Zhao M., Ma W., Wang Z., Zhang W., Yan X., Yang J. (2014). Producing more grain with lower environmental costs. Nature.

[28] Cassman K.G., Dobermann A., Walters D.T. (2002). Agroecosystems, nitrogen-use efficiency, and nitrogen management. Ambio.

[29] Guo B.Y., Gao H., Tang C., Liu T., Chu G.X. (2015). Response of water coupling with N supply on maize nitrogen uptake, water and N use efficiency, and yield in drip irrigation condition. Chin. J. Appl. Ecol..

[30] Lai Z., Fan J., Yang R., Xu X., Liu L., Li S., Zhang F., Li Z. (2022). Interactive effects of plant density and nitrogen rate on grain yield, economic benefit, water productivity and nitrogen use efficiency of drip-fertigated maize in northwest China. Agric. Water Manag..

[31] Xu C., Huang S., Tian B., Ren J., Meng Q., Wang P. (2017). Manipulating planting density and nitrogen fertilizer application to improve yield and reduce environmental impact in Chinese maize production. Front. Plant Sci..

[32] Shi D.Y., Li Y.H., Zhang J.W., Liu P., Zhao B., Dong S.T. (2016). Increased plant density and reduced N rate lead to more grain yield and higher resource utilization in summer maize. J. Integr. Agric..

[33] Guo X., Liu W., Yang Y., Liu G., Ming B., Xie R., Wang K., Li S., Hou P. (2025). Matching the light and nitrogen distributions in the maize canopy to achieve high yield and high radiation use efficiency. J. Integr. Agric..

[34] Wei S., Wang X., Li G., Qin Y., Jiang D., Dong S. (2019). Plant density and nitrogen supply affect the grain-filling parameters of maize kernels located in different ear positions. Front. Plant Sci..

[35] Ciampitti I.A., Murrell S.T., Tuinstra M., Camberato J.J., Xia Y., Friedemann P., Vyn T.J. (2013). Physiological dynamics of maize nitrogen uptake and partitioning in response to plant density and N-stress factors: II. Reproductive phase. Crop Sci..

[36] Mueller S.M., Vyn T.J. (2016). Maize plant resilience to N stress and post-silking N capacity changes over time: A review. Front. Plant Sci..

[37] Ciampitti I.A., Vyn T.J. (2013). Grain nitrogen source changes over time in maize: A review. Crop Sci..

[38] Zhai C., Zhou H.P., Zhao J. (2017). Experimental study on inter-annual water requirement and water consumption of drip irrigation maize in north of Xinjiang. Sci. Agric. Sin..

[39] Borrás L., Slafer G.A., Otegui M.E. (2004). Seed dry weight response to source–sink manipulations in wheat, maize and soybean: A quantitative reappraisal. Field Crops Res..

[40] Rajcan I., Tollenaar M. (1999). Source:sink ratio and leaf senescence in maize: II. Nitrogen metabolism during grain filling. Field Crops Res..

[41] Mi G., Liu J., Chen F., Zhang F., Cui Z., Liu X. (2003). Nitrogen uptake and remobilization in maize hybrids differing in leaf senescence. J. Plant Nutr..

[42] Kosgey J.R., Moot D.J., Fletcher A.L., McKenzie B.A. (2013). Dry matter accumulation and post-silking N economy of ‘stay-green’ maize (*Zea mays* L.) hybrids. Eur. J. Agron..

[43] Chen Y., Xiao C., Chen X., Li Q., Zhang J., Chen F., Yuan L., Mi G. (2014). Characterization of the plant traits contributed to high grain yield and high grain nitrogen concentration in maize. Field Crops Res..

[44] Bao S.D. (2000). Soil and Agricultural Chemistry Analysis.

[45] Ritchie S.W., Hanway J.J., Benson G.O. (1986). How a Corn Plant Develops.

[46] Montgomery E.G. (1911). Correlation studies in corn. Neb. Agric. Exp. Stn. Annu. Rep..

[47] Moll R.H., Kamprath E.J., Jackson W.A. (1982). Analysis and interpretation of factors which contribute to efficiency of nitrogen utilization. Agron. J..

[48] Raun W.R., Johnson G.V. (1999). Improving nitrogen use efficiency for cereal production. Agron. J..

