# Supplementary material for: Enhanced Nitrogen Supply Sustains Post-Silking Canopy Function and Grain Yield of Drip-Irrigated Spring Maize Under Increased Planting Density in Northern Xinjiang
Source: Plants (Basel). 2026 Nov 6;15(21):3467. doi: 10.3390/plants15213467 (PMC13765634; doi:10.3390/plants15213467)
Supplement: Supplementary file 1 [file plants-15-03467-s001.zip › plants-4650726-supplementary.pdf]

Supplementary Materials

Enhanced Nitrogen Supply Sustains Post-Silking Canopy Function and Grain Yield of Drip-Irrigated Spring Maize Under Increased Planting Density in Northern Xinjiang

Qi Xu, Changkun Ma, Quanjiu Wang and Qiuping Fu

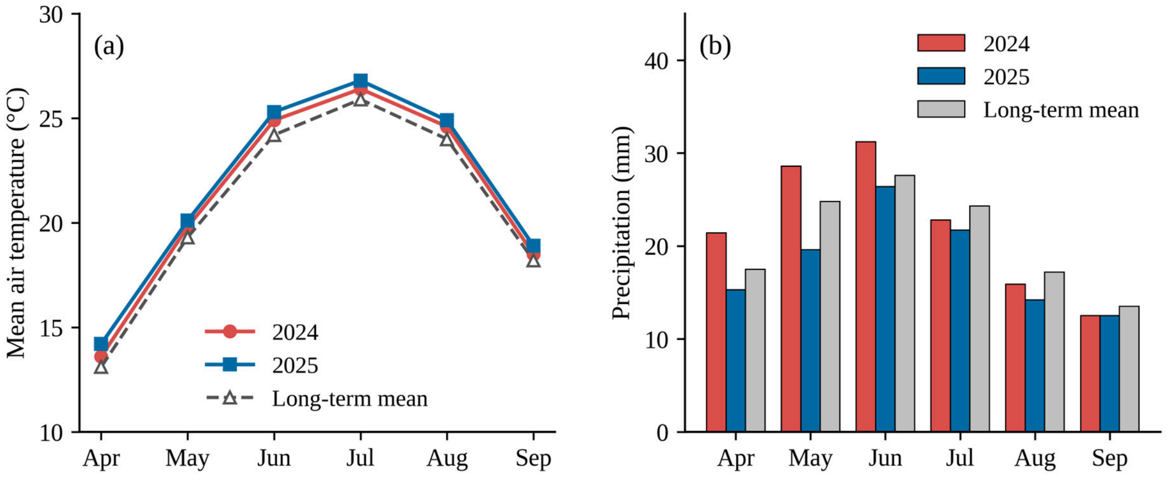

Figure S1. Monthly mean air temperature (a) and monthly precipitation (b) from April to September in 2024 and 2025 and the long-term means at the experimental site. The 2024 and 2025 data were recorded by the automatic weather station at the site; the corresponding values are listed in Table S1. Irrigation (450 mm during the season plus 30 mm after sowing) is not included.

Table S1. Monthly mean air temperature and precipitation during the maize growing seasons of 2024 and 2025 and the long-term means at the experimental site.

Month	Tmean 2024 (°C)	Tmean 2025 (°C)	Tmean LT (°C)	P 2024 (mm)	P 2025 (mm)	P LT (mm)
April	13.6	14.2	13.1	21.4	15.3	17.5
May	19.8	20.1	19.3	28.6	19.6	24.8
June	24.9	25.3	24.2	31.2	26.4	27.6
July	26.4	26.8	25.9	22.8	21.7	24.3
August	24.6	24.9	24.0	15.9	14.2	17.2
September	18.5	18.9	18.2	12.5	12.5	13.5
April–September	21.3	21.7	20.8	132.4	109.7	124.9

Tmean, monthly mean air temperature; P, monthly precipitation; LT, long-term mean. The last row gives the mean temperature and total precipitation for April–September. Irrigation (450 mm during the season plus 30 mm after sowing) is not included.

Table S2. *F* values from the split-plot analysis of variance for leaf area index (LAI) at the sixth-leaf (V6), twelfth-leaf (V12), and milk (R3) stages and for the intercellular CO_2 concentration (C_i) of the ear leaf at silking (R1) and R3 in 2024 and 2025.

Source of variation	df	LAI-V6	LAI-V12	LAI-R3	C_i-R1	C_i-R3
Year (Y)	1	0.94 ns	2.31 ns	1.73 ns	1.26 ns	2.05 ns
Density (D)	1	132.60 **	384.20 **	297.50 **	46.30 **	38.40 **
Nitrogen (N)	1	0.36 ns	1.85 ns	71.40 **	18.70 **	136.80 **
Y×D	1	0.12 ns	0.47 ns	0.58 ns	0.39 ns	0.71 ns
Y×N	1	0.05 ns	0.33 ns	0.96 ns	0.22 ns	0.48 ns
D×N	1	0.21 ns	0.62 ns	18.90 **	6.02 *	22.60 **
Y×D×N	1	0.08 ns	0.14 ns	0.27 ns	0.09 ns	0.31 ns

Year was tested against replicate (year) (df = 4); D and Y × D against the main-plot error (df = 4); other effects against the subplot error (df = 8). * and ** indicate significance at $p < 0.05$ and $p < 0.01$, respectively; ns, not significant. Results for LAI at R1 and R6 and for P_n, g_s, and SPAD value are given in Table 1b of the main text.

Table S3. Pearson correlation coefficients between grain yield and yield components, canopy and leaf traits, and dry matter and N traits calculated from pooled plot-level data (n = 24; as in Figure 4) and partial correlation coefficients after removing the effects of year, density, N rate, and their interactions.

Trait	Pooled r (n = 24)	Partial r (df = 15)
Ear number (EN)	0.78 **	0.36 ns
Kernels per ear (KNE)	−0.53 **	0.22 ns
1000-kernel weight (TKW)	−0.34 ns	0.41 ns
LAI at R3 (LAI-R3)	0.93 **	0.45 ns
P_n at R3 (P_n-R3)	−0.29 ns	0.29 ns
SPAD value at R3 (SPAD-R3)	−0.20 ns	0.18 ns
Post-silking DM accumulation (PSDM)	0.94 **	0.68 **
Pre-silking DM translocation (DMT)	−0.12 ns	0.12 ns
Post-silking N uptake (PSNU)	0.67 **	0.43 ns
Total N accumulation at maturity (Nt)	0.93 **	0.57 *

Partial correlation coefficients were calculated from the residuals of the three-way factorial model (year × density × N rate), i.e., after removing the variation among the eight year–treatment combinations; they therefore describe plot-to-plot co-variation within treatments. Critical values of $|r|$ are 0.404 ($p < 0.05$) and 0.515 ($p < 0.01$) for the pooled coefficients (df = 22) and 0.482 and 0.606 for the partial coefficients (df = 15). PFPN is not included because, within an N rate, PFPN is proportional to grain yield (partial r = 1 by definition). * and ** indicate significance at $p < 0.05$ and $p < 0.01$, respectively; ns, not significant.
